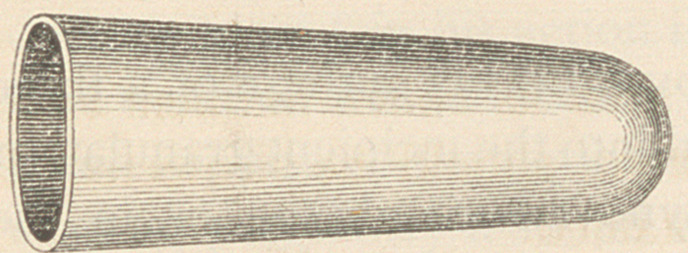# Leucorrhœa of Pregnancy

**Published:** 1882-02

**Authors:** Wm. F. Waugh


### LEUCORRHŒA OF PREGNANCY.

Although leucorrhœa is merely a symptom, and not in itself a disease, it is frequently the principal point upon which our therapeutics are brought to bear. Dependent upon widely different pathological conditions, it may in any case call for treatment to check the flow.

Every physician who treats this class of disease knows how well he is repaid for the systematic investigation of each case; and what light is thus shed upon its treatment. But such an examination is not to be made in unmarried females, unless treatment of rational symptoms fails; and in some other conditions it is contra-indicated formally.

Especially is this true of the leucorrhœa of pregnancy. Here we must lay aside the probe, use the speculum with caution, and may even find the touch difficult if the vagina be abnormally sensitive.

Very often the leucorrhœa is due to gonorrhœa, which is peculiarly apt to be contracted at this period. Catarrh of the cervix is responsible for many cases; and less frequently it is due to relaxation. The ordinary means of treatment are contraindicated. Injections, hot or cold, are unsafe. So, also, are irritant remedies; while the medicated cotton tampon, so valuable at other times, may cause perilous irritation in pregnacy.

The condition of the patient is one of great distress, especially in gonorrhœal cases, where the discharge scalds the vulva and thighs, causing intolerable torment.

To the infant there is the grave danger of ophthalmia neonatorum, from contact with the discharge.

For this condition we have had of late a most valuable therapeutical resource offered to us, in hollow vaginal suppositories now in market, manufactured by Dr. C. L. Mitchell, Ninth and Race streets, Philadelphia.

The old suppository of cocoa butter has seen its day. It was filthy and inefficient, quickly melting, while it could not be retained anywhere but just within the vaginal sphincter.

The new preparations (of which the above cut represents the exact size) are composed of gelatine and glycerine (variously medicated), cast in a hollow form, and inserted on a pledget of cotton. They melt slowly, thus medicating the vagina much longer than the cocoa butter.

They are light, and can be readily retained in the upper part of the vagina, while the heavier ones would sink.

Until the suppository is melted, the cotton tampon will not irritate the womb; and when it does, it can be at once withdrawn. Their cleanliness is a recommendation with the patients, which is of value.

It is highly probable, as claimed, that absorption takes place more readily than when the medicine is enveloped in an unctuous material. For these reasons, and especially from the length of time the remedy is kept in contact with the mucous membrane, it follows that this is the most effectual method of treating leucorrhœa and gonorrhœa yet proposed to the medical public.

I have lately treated three cases of leucorrhœa, two cases of them gonorrhœa, in pregnant women. The formula I prefer is that which bears the name of Dr. Parker, of Plymouth, Mass. Each suppository contained 10 grs. sulpho carbolate of zinc, and ½ gr. sulphate of morphia. In each case the cure was rapid, safe, and permanent. My patients were pleased with the quick relief following new treatment, and in no case was there any irritation of the uterus. One of the gonorrhœal cases has since finished the pregnancy, and the child has had no ophthalmia. The others have, as yet, had no return of the leucorrhœa.—Wm. F. Waugh, in *Med. and Surg. Reporter.*

## Figures and Tables

**Figure f1:**